# Complementary approaches to dissect late leaf rust resistance in an interspecific raspberry population

**DOI:** 10.1093/g3journal/jkae202

**Published:** 2024-08-22

**Authors:** Melina Prado, Allison Vieira da Silva, Gabriela Romêro Campos, Karina Lima Reis Borges, Rafael Massahiro Yassue, Gustavo Husein, Felix Frederik Akens, Marcel Bellato Sposito, Lilian Amorim, Pariya Behrouzi, Daniela Bustos-Korts, Roberto Fritsche-Neto

**Affiliations:** Department of Genetics, Luiz de Queiroz College of Agriculture/University of São Paulo, Piracicaba 13418-900, Brazil; Department of Genetics, Luiz de Queiroz College of Agriculture/University of São Paulo, Piracicaba 13418-900, Brazil; Department of Genetics, Luiz de Queiroz College of Agriculture/University of São Paulo, Piracicaba 13418-900, Brazil; Rice Research Station, Louisiana State University, Baton Rouge, LA 70803, USA; GDM Seeds, Campinas 13092-599, Brazil; Department of Genetics, Luiz de Queiroz College of Agriculture/University of São Paulo, Piracicaba 13418-900, Brazil; Biometris, Wageningen University and Research, Wageningen 6708 PB, Netherlands; Department of Genetics, Luiz de Queiroz College of Agriculture/University of São Paulo, Piracicaba 13418-900, Brazil; Department of Genetics, Luiz de Queiroz College of Agriculture/University of São Paulo, Piracicaba 13418-900, Brazil; Biometris, Wageningen University and Research, Wageningen 6708 PB, Netherlands; Facultad de Ciencias Agrarias y Alimantarias, Universidad Austral de Chile, Valdivia 5090000, Chile; Department of Genetics, Luiz de Queiroz College of Agriculture/University of São Paulo, Piracicaba 13418-900, Brazil; Rice Research Station, Louisiana State University, Baton Rouge, LA 70803, USA

**Keywords:** *Rubus sp*, *Acculeastrum americanum*, GWAS, copula graphical models network, high-throughput phenotyping, Plant Genetics and Genomics

## Abstract

Over the last 10 years, global raspberry production has increased by 47.89%, based mainly on the red raspberry species (*Rubus idaeus*). However, the black raspberry (*Rubus occidentalis*), although less consumed, is resistant to one of the most important diseases for the crop, the late leaf rust caused by *Acculeastrum americanum* fungus. In this context, genetic resistance is the most sustainable way to control the disease, mainly because there are no registered fungicides for late leaf rust in Brazil. Therefore, the aim was to understand the genetic architecture that controls resistance to late leaf rust in raspberries. For that, we used an interspecific multiparental population using the species mentioned above as parents, 2 different statistical approaches to associate the phenotypes with markers [GWAS (genome-wide association studies) and copula graphical models], and 2 phenotyping methodologies from the first to the 17th day after inoculation (high-throughput phenotyping with a multispectral camera and traditional phenotyping by disease severity scores). Our findings indicate that a locus of higher effect, at position 13.3 Mb on chromosome 5, possibly controls late leaf rust resistance, as both GWAS and the network suggested the same marker. Of the 12 genes flanking its region, 4 were possible receptors, 3 were likely defense executors, 1 gene was likely part of signaling cascades, and 4 were classified as nondefense related. Although the network and GWAS indicated the same higher effect genomic region, the network identified other different candidate regions, potentially complementing the genetic control comprehension.

## Introduction

Although wild raspberry species occur in different conditions, production is mainly in Europe and North America. With a production increase of 47.89% in 10 years (FAOSTAT 2022), this temperate crop is increasingly appreciated due to several characteristics. Its main features are organoleptic properties, content of healthy components, such as anthocyanins (Rao and Snyder 2010), and high added value, which can represent a potential business for small and medium-sized producers (de Oliveira *et al.* 2020). Even with this set of characteristics, there is a need for more raspberry cultivars adapted to tropical climate conditions, as is the case in Brazil. In Brazil, the used varieties are introgressions from north-hemisphere countries (Marchi *et al.* 2019), so their yield and performance in the face of biotic and abiotic stresses depend on the intensity of the interaction of these cultivars with the new production environments. The country’s production is concentrated in regions with high altitudes and lower annual temperatures, representing a small portion of arable areas of Brazil.

The acceptance of a new raspberry cultivar relies on environmental adaptation and fruit quality, with particular emphasis on fruit color. Red raspberries (*Rubus idaeus*) are economically more important and more consumed than black ones (*Rubus occidentalis*) (Folta and Gardiner 2009; Baby *et al.* 2018). However, in addition to the differences in fruit color, they also differ in resistance to some diseases. Late leaf rust is relevant in the north and south hemispheres, with reported losses of up to 70% with the cultivar “Festival” in Nova Scotia (Ellis *et al.* 1991). The *Acculeastrum americanum* pathogen causes the disease, and the first symptom is the orange spots on the leaves, which turn dark over time. Depending on the disease severity, premature defoliation may occur, consequently becoming more susceptible to winter injuries. Although most red raspberry cultivars are susceptible to late rust and are economically important, more resistant varieties, such as the black raspberry species, have been reported and may serve as a source of genetic variability (Hall *et al.* 2009). Therefore, exploring the resistance interspecific variation to late leaf rust in raspberries represents great potential for breeding and a more sustainable and indicated way to control the disease.

The different genetic variations in these species derive from spontaneous mutations maintained or shaped during evolution by some forces, such as natural or artificial selection, among other genetic processes that lead to speciation. The analysis of these mutations helps to elucidate the genetic architecture of traits of great interest to humanity, such as yield or resistance to biotic and abiotic stresses, among many others (Alonso-Blanco *et al.* 2009). Understanding complex traits requires a study of the causal loci allelic variation. The general way to make this link between phenotype and genotype is by performing broadly used analyses, genome-wide association studies (GWAS), and quantitative trait locus (QTL) mapping (Korte and Farlow 2013). The wide use of these techniques was possible due to the advancement of next-generation sequencing technologies, exponentially increasing the capacity to obtain genomic data with a drop in the associated cost (Henson *et al.* 2012). However, phenotyping remains a bottleneck for these traits studied nowadays (Yang *et al.* 2020). Our ability to dissect the genetic architecture of a quantitative trait is limited by our ability to obtain individual phenotypic values, such as traditional or high-throughput phenotypes. As traditional methods can be costly, time-consuming, destructive, and more prone to human error, high-throughput phenotyping has come to try to reduce the phenotyping bottleneck in breeding programs (Gill *et al.* 2022). Although it still presents many limitations and challenges, once established in a crop, it can accelerate genetic gain by improving the selection intensity and accuracy (Araus *et al.* 2018).

In the past years, GWAS has been the main analysis used to study the genetic architecture of traits due to its ability to control population structure and not require a specific mating design as QTL mapping. The main contribution of the technique derives from the fact that it is possible to perform these associations in panels containing great diversity. The method uses the ancestral recombination events to identify the causal loci through the linkage disequilibrium (LD) (Huang and Han 2014). The studied traits can be simple and easily detectable by GWAS when they have few loci with large effects on the phenotype or more complex traits, such as those that are regulated by many small-effect loci or those that have many rare allelic variants (Korte and Farlow 2013).

Not as conventional as association and QTL mapping in plant breeding, it is the use of a graphical network. The term *Network* has become more frequent in many scientific fields because graphical modeling is one of the best ways to represent high-dimensional data (Purutçuoğlu and Farnoudkia 2017). Behrouzi and Wit (2019) studied epistatic selection using semiparametric penalized copula Gaussian graphical models, in which the network was able to capture aberrant associations between markers through short- and long-range LD dependencies. Zhang *et al.* (2021), also using latent Gaussian copula models, studied schizophrenia using genomic data based on single nucleotide polymorphism (SNPs) and brain imaging. In these systems, each variable, such as phenotype and genotype, is represented by a node, and edges between these nodes represent its conditional independence relationships. According to Sklar’s theorem, it is necessary to describe the joint probability distribution function to use multivariate stochastic models. This joint distribution can be decomposed into the variable’s marginal distributions and a joint behavior of the random variables, the copula (Durante *et al.* 2013). Besides the capability to utilize data with missing values, a key benefit lies in the separate and independent variable modeling, allowing the joint use of variables with different distributions. As association mapping and the network derived from copula graphical models are approaches that differ in methodology and data modeling, using them together can contribute as an additional layer of reliability to the obtained results.

Given the above, this work seeks to find the genomic regions responsible for resistance to late leaf rust in raspberries using traditional and high-throughput phenotyping data through a nonconventional pipeline using a GWAS and copula graphical models network.

## Materials and Methods

### Plant material

**Fig. 1.**
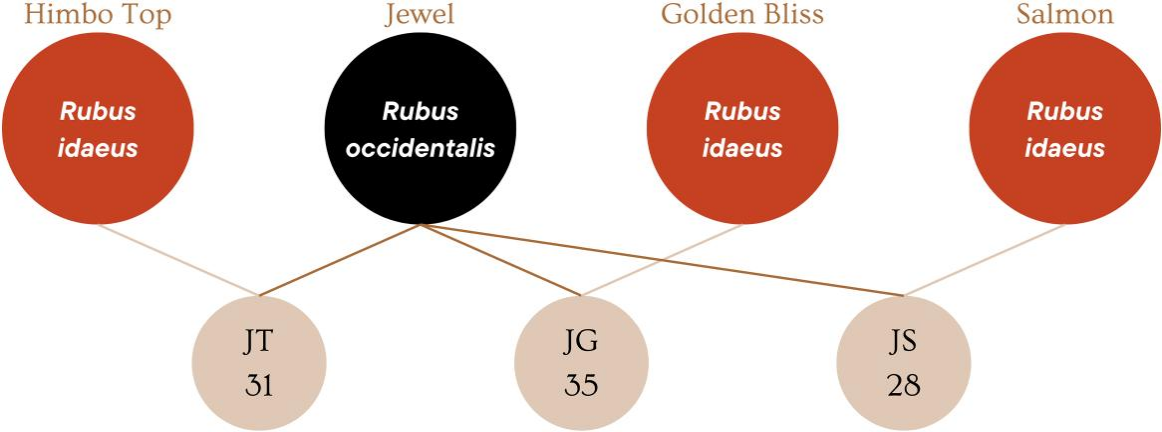
Population structure. In red (Himbo Top, Golden Bliss, and Salmon) are the varieties of *R. idaeus*, with different susceptibility levels. In black (Jewel) is the crosses common parent *R. occidentalis*, mother of all crosses, with higher resistance to late leaf rust. In beige are the subfamilies with their specific numbers of individuals.

The crosses to obtain the interspecific hybrids were carried out in a *testcross* scheme, in which a group consisted of 3 parents of the species *R. idaeus* that had favorable characteristics for the market, such as fruit color ranging from golden (Golden Bliss) to red (Himbo Top), were crossed with the common parent of the species *R. occidentalis* (Jewel), being the source of alleles for late rust resistance (Fig. 1). Both the red and black raspberry varieties used in the study are diploid, although the genome size in centiMorgans (cM) of these 2 species is different, with the red variety (Heritage) having 462.7 cM (Ward *et al.* 2013) and the black variety (Jewel) having 1230.7 cM (Willman *et al.* 2022). We highlight the number of individuals per subfamily in Fig. 1, where the JG subfamily has 35 individuals, JS has 28 individuals, and JT has 31 individuals. “Jewel” was used as a female parent due to the unilateral incompatibility between the species (Lewis and Crowe 1958). Of the 99 genotypes, 94 were interspecific hybrids, and 4 were parents. Although the population is small in number, it is highly heterozygous and the previously calculated effective population size is 174 individuals (Campos *et al.* 2023). The average population heterozygosity is 0.54, while the parents of *R. idaeus* species have 0.58, and Jewel (*R. occidentalis*) reaches 0.77. More information about the genetic characterization and diversity of the panel can be found in Campos *et al.* (2023).

The variety “Jewel” is described as a vigorous, consistently productive plant with large, firm fruits, almost black color, and higher resistance to winter injuries than red raspberries (Ourecky and Slate 1973; OMAFRA 2021). The species *R. occidentalis* has late rust resistance records, but the records are not associated with the “Jewel” variety. Although we attempted to use the parent “Heritage” we were unable to obtain any viable crosses between this variety and “Jewel.” Therefore, we used it only as a check in the experiment. The “Heritage” variety has medium, reddish, and excellent quality fruits, but it is highly susceptible to late rust and needs more hours of chilling (Hall *et al.* 2009). The cultivar “Himbo Top,” which originated in Switzerland, has pale red fruit and is easy to harvest (Hauenstein 2008; OMAFRA 2021). The cultivar “Autumn Bliss,” parent of “Himbo Top,” has good productivity in Brazil and is less demanding in terms of the number of chilling hours than “Heritage” (Raseira *et al.* 2004). The “Golden Bliss” variety, or “All Gold” in the United Kingdom, has little information in the literature. It has yellow fruit and it is well adapted to the South of the state of Minas Gerais (Moura *et al.* 2012). No information about the “Salmon” variety was found in the literature.

### Conducting the experiments

The experimental units consisted of a 5 L pot containing only one plant. The experiment was arranged in an augmented block design, which was replicated twice in time. The experiment was carried out in a semicontrolled condition, in a greenhouse with temperature control and supplemental light. One clone from each parent of the red species (“Himbo Top,” “Golden Bliss,” and “Salmon”), 1 from the resistant black raspberry “Jewel” and 1 from the unrelated variety “Heritage,” were used as checks in every block. For its execution, drastic pruning was carried out, leaving only 3 buds on the stems. Because younger leaves are less susceptible to late rust, with the drastic pruning, all the plants had leaves at approximately the same development stage. The inoculum from the fungus *A. americanum* was sprayed 5 days after the pruning. The suspensions were prepared in the Department of Phytopathology of ESALQ/USP, using 50 mL of distilled water, Tween 20 (0.01%), and urediniospores of *A. americanum* collected. The suspension concentration was adjusted to 104 urediniospores/mL in a Neubauer chamber and used to spray inoculate the abaxial face of the leaves to the point of runoff. To ensure the development of the disease, the plants were covered for 24 h with a dark plastic bag to set up a humid chamber.

### Phenotyping

**Fig. 2.**
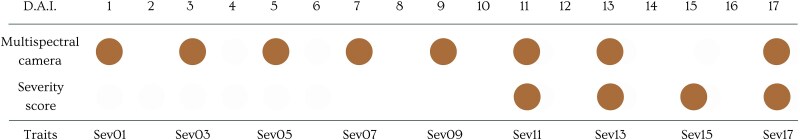
Replicate structure. The experiment was replicated twice, and there were 17 days of phenotyping after inoculation in each replicate. We phenotyped in 2 ways: traditional phenotyping using disease severity scores and high-throughput phenotyping using the multispectral camera.

The experiment consisted of 2 types of phenotyping, traditional and high-throughput phenotyping, lasting ∼17 days after inoculation (DAI) (Fig. 2). In traditional phenotyping, disease severity scores (ranging from 0 to 8) were assigned from the 11th to the 17th DAI. According to the diagrammatic scale proposed by Dias *et al.* (2023a). Since there is subjectivity related to the score given by the evaluator and to reduce this bias, 3 people evaluated all the plants and an average of the evaluators was performed.

**Fig. 3.**
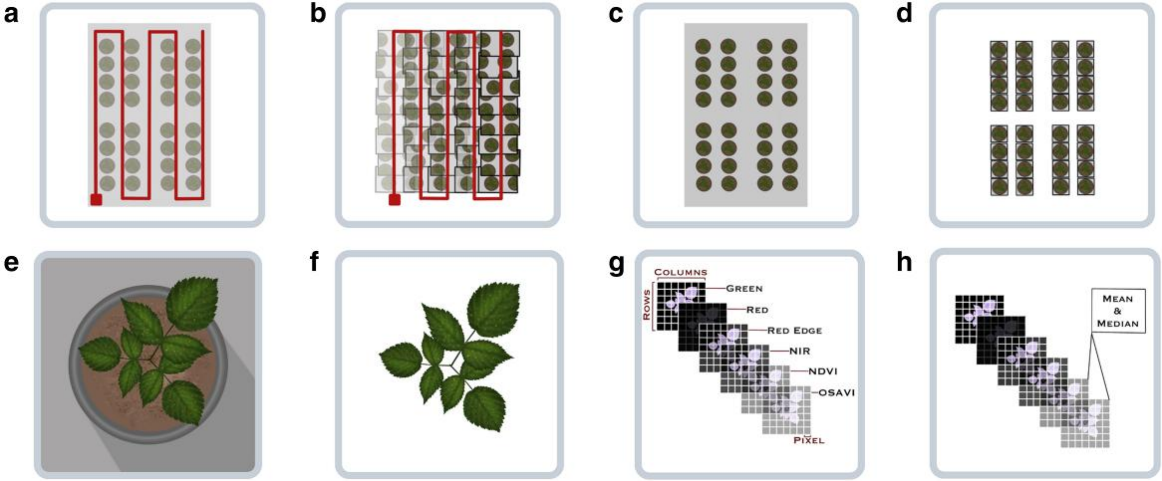
High-throughput phenotyping methodology. a) Mimicry of a drone flight inside the greenhouse using motorized rails and the multispectral camera. b) Georeferenced images with 70% overlapping. c) The orthomosaic assembly. d) The extraction of ShapeFiles from orthomosaic (pots). e) The image of a plot with the background. f) The image of a plot after removing the background. g) This same image is represented with a 3D matrix, where the third dimension represents the original and calculated layers (masks). h) The spectral indices (phenotypes) are calculated through the mean and median of NDVI and OSAVI layers.

A high-throughput greenhouse phenotyping platform obtained the plant’s multispectral images (Fig. 3). The platform consists of 3 rails, 2 moving in parallel and 1 perpendicular to the side of the greenhouse. A board, cameras, battery, and sensors were attached to the perpendicular rail. More details about this low-cost, greenhouse-based, high-throughput phenotyping platform in Yassue *et al.* (2022).

The high-throughput images were collected from the first DAI and then on alternate days until completing 17 DAI. We experienced issues capturing images on the 15th day of phenotyping, so there is no high-throughput phenotyping data for this day (Sev15). To facilitate discussion in the paper, we will design these traits as Sev01–Sev17, specifying the 1st to the 17th phenotyping DAI for disease severity.

### Image processing

The multispectral camera was the *Parrot’s Sequoia+*, with a resolution of 1.2 MP in each of the 4 monochromatic lenses: green, red, red edge, and near-infrared. The monochrome lens of the green has a wavelength of 550 nm, the red has a wavelength of 660 nm, the red edge of 735 nm, and the near-infrared of 790 nm. The images were processed by assembling the orthomosaics using the *software AgiSoft Metashape v. 1.7*. After assembly, the plots *ShapeFiles* were drawn in the *software QGIS 3.0*. To remove the background, we first apply the *Soil Color Index* (SCI) filter for soil removal with the software *FieldImageR* (Matias *et al.* 2020). As the image’s background was uneven, we had to manually adjust the intervals of each layer to remove as much of the background as possible. The spectral indices referring to the presymptomatic and symptomatic plants were extracted using the same software. We tested 2 different masks, the NDVI and OSAVI, from which we extracted the 2 indices commonly used in works studying disease severity. We used the mask layers’ mean and median, as carried out by Yassue *et al.* (2022) and as represented in Fig. 3 (step h).

### Genotyping

Genotyping was performed using the genotyping-by-sequencing (GBS) methodology (Elshire *et al.* 2011). Young fresh leaves were sampled from each genotype and immediately frozen in liquid nitrogen. These samples were stored at ${-80}^{\circ }$C until further analysis. The extraction of total DNA from each sample was carried out following the protocol proposed by the manufacturer of the extraction kit (*Qiagen*) and using the *DNeasy Plant Mini Kit*. A rare-cutting enzyme, the PstI (New England BioLabs Inc.), and another frequently cutting enzyme, the MseI (New England BioLabs Inc.), were used. Purification with the *QIAquick PCR purification Kit* (*Qiagen*) was followed to proceed with the PCR amplification step. DNA libraries were quantified using the *Agilent DNA 1000 Kit* in an *Agilent 2100 Bioanalyzer* (Agilent Technologies). Additionally, they were quantified on a *CFX 384 Touch Real-Time PCR Detection System* using the *KAPA Library Quantification* Kit (*KAPA Biosystems, cat. KK4824*). Finally, the libraries were diluted and sequenced in a sequencer *HiSeq 2500 System* (*Illumina, Inc*). The SNP calling was carried out following the pipeline *TASSEL-GBS* (Glaubitz *et al.* 2014). The alignment was performed using *Bowtie2 software* (Langmead and Salzberg 2012) with the reference genomes of the black raspberry (VanBuren *et al.* 2016). We started our analysis with the 28.373 markers previously selected in the work of Campos *et al.* (2023), but to perform filtering and imputation on the data, we use these markers in their original format containing missing values. Filters, such as minor allele frequency ≥0.20, missing rate ≤0.20, and a filter to remove monomorphic SNPs, were applied to remove possible sequencing errors using the package *SNPRelate* (Zheng *et al.* 2023). We used this MAF value because it helped to control the large variability in the panel and improve the resolution of the GWAS in our small population and because we were interested in the resistance alleles of the central parent. Of the initial 28.373 markers, we ended with 19.440 markers after filtering. Finally, the snpReady software was used for the missing data imputation by Wright’s method (Granato *et al.* 2018).

### Phenotypic analysis

The genotypic values and variance components were estimated using the REML/BLUP method (restricted maximum likelihood/best linear unbiased predictor) using the *statgenSTA* package (Rossum *et al.* 2023) in the R environment (version 4.3, https://www.r-project.org/). The following model was used to calculate the genotypes BLUPs and to estimate the variance components and broad-sense heritabilities:


(1) $$y=X_{1}r+X_{2}c+Z_{1}b+Z_{2}g+\epsilon$$

Where y is the vector of phenotypic values of disease severity; r is the replicate fixed effect; c is the checks fixed effect; b is the random replicate nested block effect, where $N(0,I\sigma_{b}^{2})$; g is the random genotype effect, where $N(0,I\sigma_{g}^{2})$; ϵ is the random effect of the residual, where $N(0,I\sigma_{e}^{2})$; $X_{1}$ and $X_{2}$ are the fixed effect incidence matrices; $Z_{1}$ and $Z_{2}$ are the random effect incidence matrices; and I is the identity matrix. BLUPs were considered in the next steps because it was necessary to perform a spatial correction using checks with the *statgenSTA* package, due to the heterogeneity of spatial effects and the experimental design in the greenhouse. As *statgenSTA* package uses *SpATS* package as a modeling engine, an extra spatial term is always included in the model. The P-spline ANOVA (PSANOVA) uses penalized bidimensional splines to adjust local variations using the column and row coordinates of the experimental data (Rodríguez-Álvarez *et al.* 2017).

### Genome-wide association studies

From model (1), association mapping was performed using the Bayesian information and linkage disequilibrium iteratively nested keyway (BLINK) method, implemented in the *GAPIT software* (Lipka *et al.* 2012). As BLINK model accounts for population structure as it uses putative quantitative trait nucleotides (QTNs) as covariates in the model (Chen *et al.* 2021), we did not use any principal components as covariates in the model. Quantile-quantile and Manhattan plots were generated to scan the population stratification and to visualize the significant SNPs in the association mapping analysis. To calculate the significance threshold, we used an adjusted *P*-value for the effective number of tests in our study by the analysis of LD blocks across the genome (Johnson *et al.* 2010). According to Campos *et al.* (2023), the average gene block for our population is 282 kb. Dividing the size of the black raspberry genome (243 Mb) by the average size of the gene blocks, we obtained a number of 861 gene blocks in our genome. Then, we divided the alpha value of 0.05 by the effective number of tests (number of gene blocks). Therefore, we corrected the *P*-value to 5.8e−05 and considered a new threshold of 4.23 on the negative logarithmic scale.

### Copula graphical model

The *netgwas* package was used to estimate the conditional independence relationships with a nonparanormal (npn) approach within the Gaussian copula graphical model (Behrouzi *et al.* 2023), this method was chosen because of the data high dimensionality. We built ten networks with different sparsity levels from the following “rho” settings: 0.1, 0.2, 0.25, 0.3, 0.4, 0.5, 0.6, 0.65, 0 .7, 0.75. After building the 10 networks, we used a package function that calculates the best regularization parameter (rho) based on Bayesian criteria, which was 0.2 for our data. As input for the network, we used a genotype by variable matrix, with the variables being all the BLUPs from the model (1) and all markers. Since copula graphical models are computationally intensive, we reduced the variables number. The *SNPRelate* package was used to filter the marker matrix by LD, using a sliding window with a size of 100 kb and an LD threshold of 0.10, resulting in a 4,686 markers matrix (Zheng *et al.* 2023). We ensured that the SNPs found by GWAS were in this new marker matrix, so we could compare the results. To facilitate the discussion of the resulting network, we adopted a partial correlation threshold of 0.05 between the markers and the traditional phenotypes (Sev11–Sev17). Finally, we used the *netShiny* package to graphically visualize the network (de Jongh and Behrouzi 2022).

### Gene annotation

All candidate genes flanking the associated marker were analyzed considering a 100 kb window, or 50 kb upstream and downstream regions of the physical positions of the associated SNPs. These genes were annotated by using the *R. occidentalis* reference genome. In addition to gene annotation by homology using BLAST, the conserved domains/motifs of R genes from the published *R. occidentalis* genome were predicted by the *InterProScan v5.33–72.0 software*. According to the literature, candidate genes were classified as not defense-related, belonging to the signaling cascade, as receptors or defense executors.

### SNPs heritability

To estimate the percentage that all associated SNPs explain of the phenotype, we have calculated the SNPs variance components based on the following model:


(2) $$y*=Z_{1}g+\sum_{i=1}^{\;p}Z_{i+1}s_{i}+\epsilon$$

To estimate the percentage that each associated SNP explains of the phenotype, we have calculated the SNPs variance components based on the following model:


(3) $$y*=Z_{1}g+Z_{2}s_{i}+\epsilon$$

Where y* is the vector of the disease severity BLUPs calculated in model (1); g is the random genotype effect, where $N(0,G\sigma_{g}^{2})$ and G is the additive kinship matrix; $s_{i}$ is the random effect of the ith SNP ($s_{i};i=1,\ldots ,p$), where $N(0,I\sigma_{si}^{2})$; ϵ is the random effect of the residual, where $N(0,I\sigma_{e}^{2})$; $Z_{1}$, $Z_{i+1}$, …, $Z_{\;p+1}$ are the random effect incidence matrices; and I is the identity matrix. As we were using the SNPs as random covariates in the model, we removed them in the additive G matrix.

From model (2), we calculated the narrow-sense heritability for all SNPs jointly by the following model:


(4) $${h^{2}}_{\mathrm{total}}=\frac{\sum_{i=1}^{\;p}\sigma_{si}^{2}}{\sigma_{g}^{2}+\sum_{i=1}^{\;p}\sigma_{si}^{2}+\sigma_{e}^{2}}$$

From model (3), we calculated the narrow-sense heritability for each SNP:


(5) $${h^{2}}_{\mathrm{snp}}=\frac{\sigma_{si}^{2}}{\sigma_{g}^{2}+\sigma_{si}^{2}+\sigma_{e}^{2}}$$

## Results

### Traditional traits

#### Severity score BLUPs

**Fig. 4.**
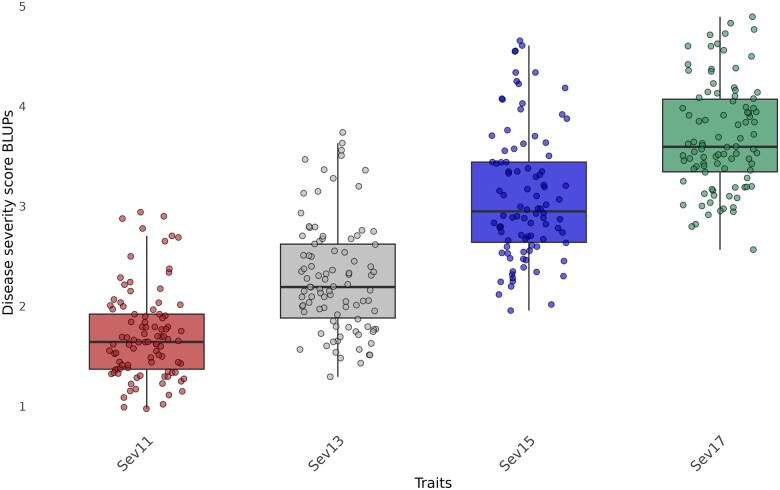
Severity score BLUPs distribution from the 11th to the 17th day after inoculation, or for Sev11–Sev17 traits.

The increase in mean disease severity score BLUPs shows that we were able to capture disease progression with the traditional phenotyping method used (Fig. 4). For the same BLUPs, the lowest correlation observed was between Sev11 and Sev17, with a correlation coefficient of 0.88. The highest correlation, 0.97, was found between the Sev15 and Sev17 traits. Broad-sense heritabilities ranged from 0.36 for Sev17 to 0.40 for Sev13 (Table 1).

**Table 1.** Pearson correlation and broad-sense heritability of disease severity BLUPs (traditional phenotyping) from the 11th to 17th DAI.

	Traits				Broad-sense heritability
	Sev11	Sev13	Sev15	Sev17	
Sev11	1				0.38
Sev13	0.96	1			0.40
Sev15	0.92	0.96	1		0.39
Sev17	0.88	0.93	0.97	1	0.36

#### Genome-wide association studies for traditional traits

**Fig. 5.**
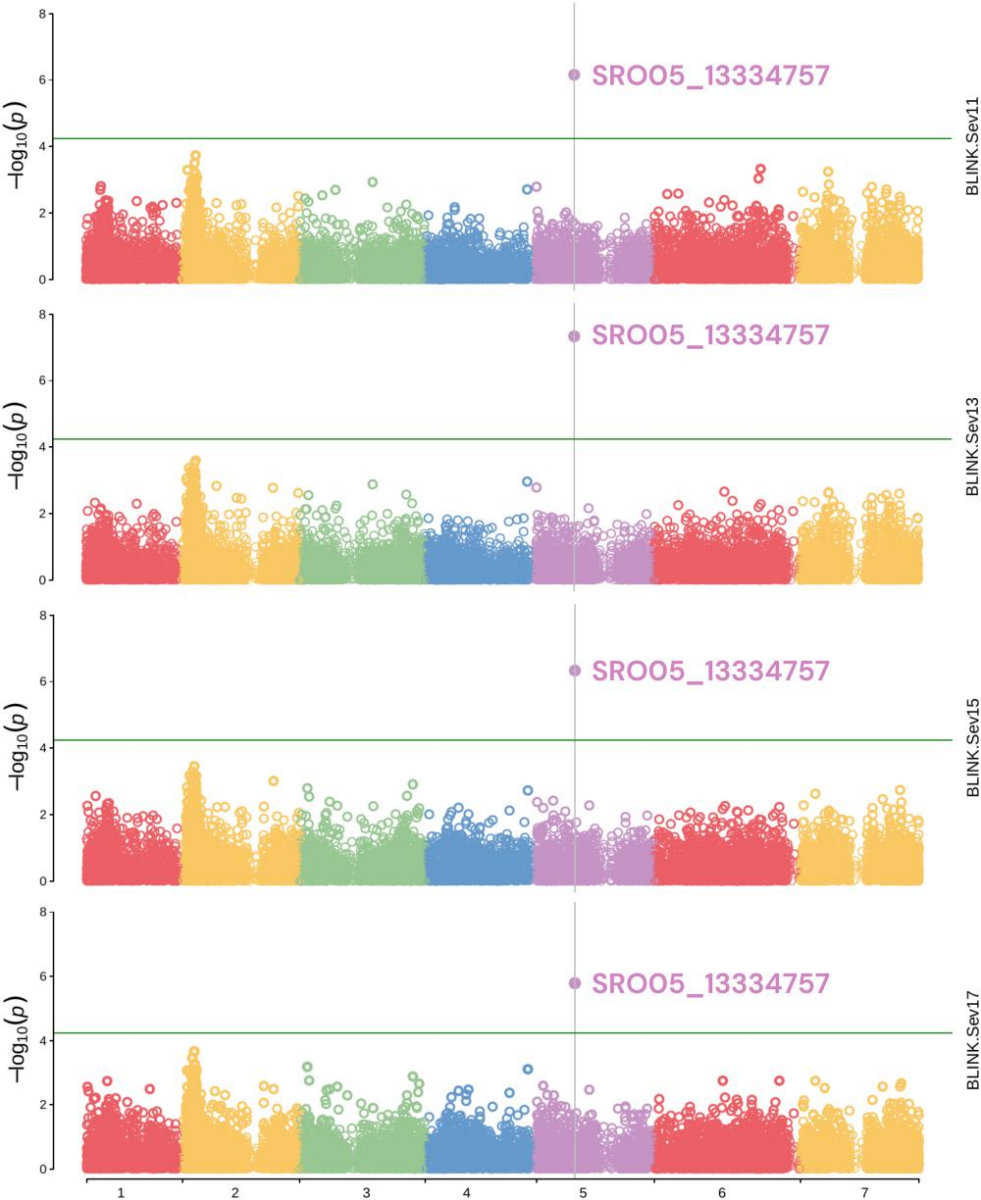
Manhattan Plots from the GWAS for each of the 4 traditional traits, Sev11–Sev17. The vertical black line represents the marker with the lowest *P*-value. The horizontal green line is the significance threshold calculated for GWAS.

We found a significant marker at position 13.3 Mb of chromosome 5 in all traditional phenotyping days (Fig. 5). A table containing the marker name, traditional phenotyping day, chromosome, position, *P*-value, maf, and SNP effect can be found as Supplementary Table S1. We already expected to find fewer SNPs because it is a small population, so we decided to monitor this SNP because it is consistent over time. To facilitate the results presentation in this section, we will comment on genes directly related to plant immunity. However, in the next sections, we will show the gene profile of all genes found by GWAS and the network (Fig. 8). A list of the entire annotation can be found in the Supplementary material for all genes flanking the marker region in a 100 kb window (Supplementary Table S2). Of the 12 genes flanking its region, 4 were possible receptors, 3 were likely defense executors, 1 gene was part of signaling cascades, and 4 were classified as nondefense related. Approximately 20 kb from this marker is a gene whose function prediction is “NB-ARC DOMAIN DISEASE RESISTANCE PROTEIN.” In the exact position of the marker, there is another gene with the function “PLANT BROAD-SPECTRUM MILDEW RESISTANCE PROTEIN RPW8.” 20 kb away, we found a receptor gene with the function “L-TYPE LECTIN-DOMAIN CONTAINING RECEPTOR KINASE S.5-RELATED.” 6.8 kb away from the marker, we found the 4th gene with receptor function, the gene “WALL-ASSOCIATED RECEPTOR KINASE-LIKE 21.” In addition to the Manhattan plots, we observed that the QQPlots are adjusted (Supplementary Fig. S1), with the majority of markers having an expected −log10(*P*-value) and only one marker with the observed −log10(*P*-value) much higher than expected, supporting the significance of this marker on chromosome 5.

#### Copula graphical models network

**Fig. 6.**
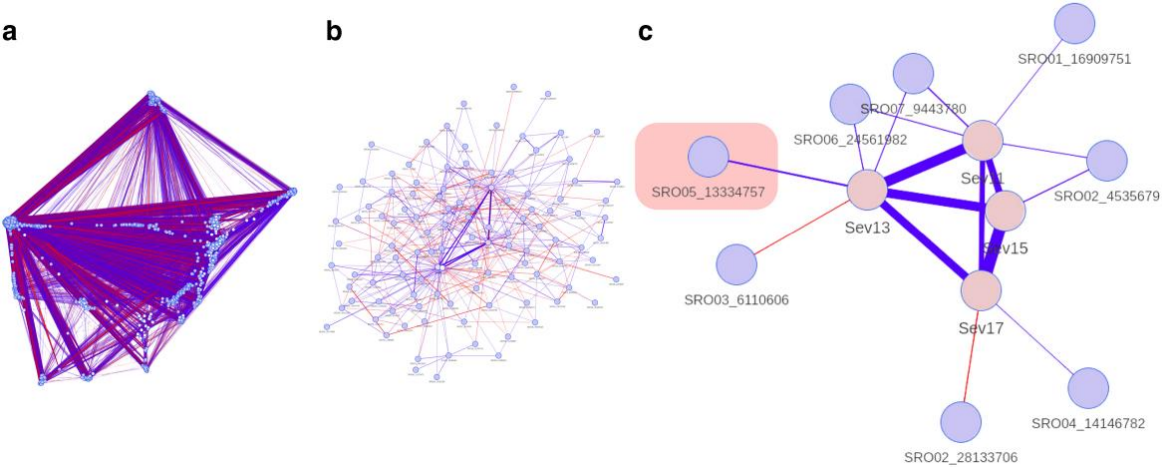
Copula graphical models networks. a) The network containing all variables; b) contains only the markers with primary dependencies on the 4 traditional traits; c) again a subnetwork containing variables with a partial correlation higher than or equal to 0.05. The pink square in c) highlights the same significant marker in GWAS.

The nodes in the network derived from copula graphical models represent all variables, including the 4 traditional traits and the 4,686 markers used (Fig. 6). The edge thickness indicates the degree of partial correlation between variables. From left to right, we present the network containing all variables (Fig. 6a), a subnet containing only the markers with primary dependencies on the 4 traditional traits (Fig. 6b), and a subnet containing variables with a partial correlation of 0.05 or higher (Fig. 6c). We selected partial correlations of at least 0.05 to facilitate the discussion of our work. In the same figure, the edges connecting the 4 traditional traits are thicker and have higher correlations compared to the correlations with the markers. As with GWAS, annotations for all associated markers are presented in Supplementary Table S2.

**Fig. 7.**
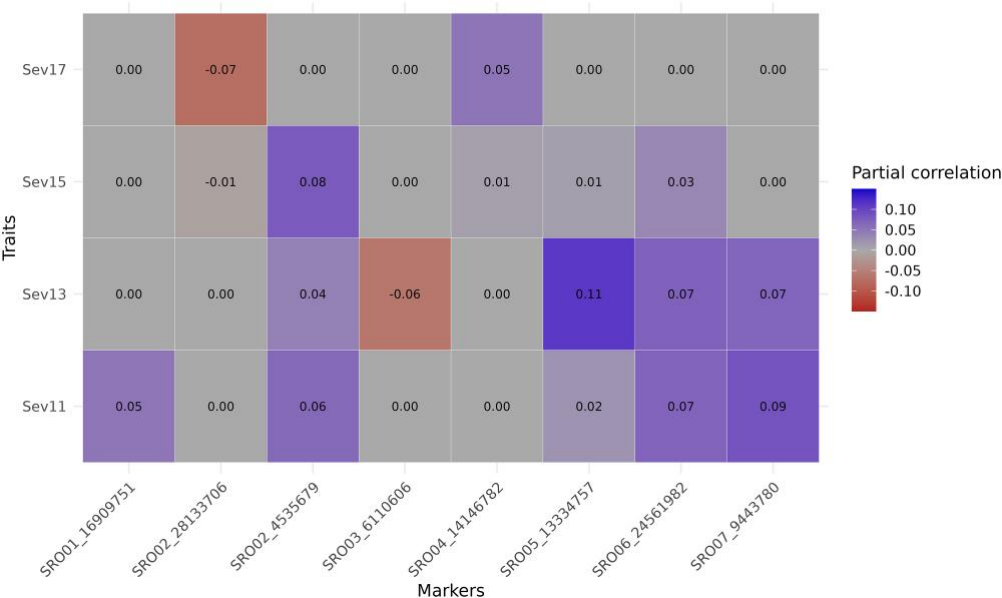
Matrix representation of partial correlations higher than 0.05, between phenotypic and marker variables. The closer the partial correlation is to blue, the more positively related the allele is to the traits. The closer the partial correlation is to red, the more negatively related the allele is to the traits. The gray color represents a partial correlation of zero between the variables.

The marker with the higher partial correlation in the network is the SRO05_13334757 marker in 13 DAI (Sev13) (Figs. 6 and 7), the same as in GWAS. But unlike GWAS, the network provided us with more complex information about the architecture of late rust resistance. We observed that the 4 phenotypic variables (in pink in Fig. 6c) are linked to at least 1 marker (in blue in Fig. 6c), and that some phenotypic variables have more than 1 marker linked to them at the same time. In the partial correlation matrix in Fig. 7, some associations are positive (blue) and others negative (red), suggesting that disease severity is negatively or positively correlated with the lack or presence of the reference allele or the most common allele at a given SNP.

#### Markers’ gene profile

**Fig. 8.**
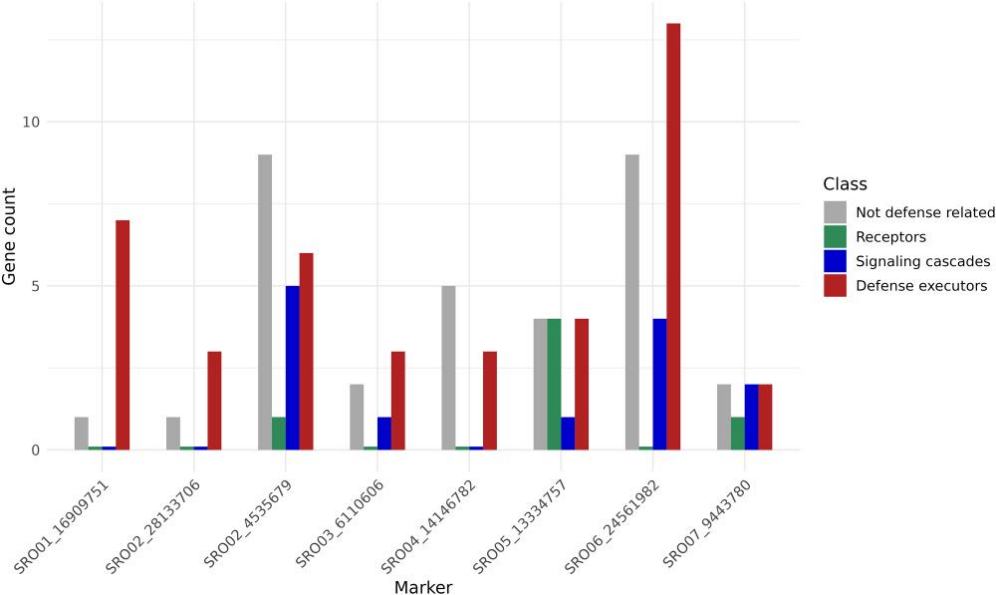
The marker’s gene profile in GWAS and the copula graphical models network. In gray (first position for each marker) is the not defense-related gene count for each marker on the *X*-axis. In green (second position) is the gene count that has a receptor function. In blue (third position) is the gene count that participates in signaling cascades. In red (fourth position) is the gene count with functions belonging to the class of defense executors genes.

The gene profile for each associated marker shows that regions flanking the 8 markers of interest had 93 genes (Fig. 8). Of this total, we classified each gene as “Not defense-related,” Receptors, “Signaling cascades,” and “Defense executors” based on the literature. Of the 93 genes, we classified 33 genes as “Not defense-related.” The marker with the highest number of “Defense Executors” in red is the SRO06_2456198. The marker with the highest number of “Receptors” in green is the SRO05_13334757. The marker with the highest number of “Signaling cascades” in blue is the SRO02_4535679.

#### Graphical network gene annotation

The 4 phenotypic variables in the network are linked to 8 different marker variables, with 93 candidate genes flanking these markers. The phenotypic variable Sev11 has partial correlations with 4 different markers (Fig. 6c). For example, these markers have candidate genes with functions such as “ankyrin repeat-containing protein NPR4,” “probable leucine-rich repeat receptor-like protein kinase At1g68400,” “probable WRKY transcription factor 2,” “pleiotropic drug resistance protein 1,” and “salicylic acid-binding protein 2.” Similarly, the Sev13 variable is linked to 4 markers, with candidate genes including “probable serine/threonine-protein kinase kinX” and all the other candidate genes mentioned for the highlighted marker on chromosome 5. Unlike the previous markers, the Sev15 phenotypic variable has only one marker linked to it, with candidate genes such as the “probable serine/threonine-protein kinase kinX” gene. Finally, the Sev17 variable has a partial correlation with 2 markers that are not common to any of the other phenotypic variables and only have genes likely to be defense executors, such as the “FAR-RED IMPAIRED RESPONSIVE (FAR1) FAMILY PROTEIN” gene (Fig. 8).

#### SNPs heritabilities

We calculated the heritability for 8 SNPs, the marker found in GWAS, and the other 7 SNPs found only by the network. The heritability resulting from the combined use of all 8 SNPs (total) for Sev11 was 0.5135, while for Sev13, Sev15, and Sev17, it was 0.5850, 0.5831, and 0.5826, respectively (Table 2). For the SRO05_13334757 marker, the $h_{\mathrm{snp}}$ on the different phenotyping days were 0.3739, 0.4313, 0.3852, and 0.3477. The SRO06_24561982 marker and the chromosome 5 marker obtained the highest heritabilities over the phenotyping days and had values of 0.3205, 0.3486, 0.3074, and 0.3013, respectively.

**Table 2.** The narrow-sense heritability for each individual SNP, coming from both the network and GWAS, and for all SNPs jointly (total) on all traditional phenotyping days.

	Sev11	Sev13	Sev15	Sev17
${h^{2}}_{\mathrm{SRO}01\_16909751}$	0.2291	0.2141	0.1492	0.1301
${h^{2}}_{\mathrm{SRO}02\_28133706}$	0.0732	0.1327	0.2356	0.2579
${h^{2}}_{\mathrm{SRO}02\_4535679}$	0.1697	0.1929	0.2084	0.1989
${h^{2}}_{\mathrm{SRO}03\_6110606}$	0.213	0.2463	0.1786	0.1358
${h^{2}}_{\mathrm{SRO}04\_14146782}$	0.0485	0.0517	0.1117	0.1719
${h^{2}}_{\mathrm{SRO}05\_13334757}$	0.3739	0.4313	0.3852	0.3477
${h^{2}}_{\mathrm{SRO}06\_24561982}$	0.3205	0.3486	0.3074	0.3013
${h^{2}}_{\mathrm{SRO}07\_9443780}$	0.3336	0.3225	0.2653	0.2165
${h^{2}}_{\mathrm{Total}}$	0.5135	0.585	0.5831	0.5826

### High-throughput phenotyping

#### Genome-wide association studies for high-throughput traits

Significant markers were found on all high-throughput phenotyping days using the GWAS approach. We noticed that both NDVI and OSAVI masks produced the same results. A complete list of all indices, with associated markers, *P*-values, correlation with traditional traits and broad-sense heritability are available as Supplementary Table S3. However, the indices had a correlation with traditional traits from 0.04 to 0.26, while heritability varied from 0 to 0.43. We annotated genes for the marker associated with the spectral index that had the highest correlation with traditional traits. This index was the mean NDVI and mean OSAVI at 11 DAI, which has a heritability of 0.26. The associated marker is positioned in 30.6 Mb at chromosome 4 and has 32 genes in a 100 Mb window. Among all these genes, 41 kb from the marker there is a gene whose predicted function is “LEUCINE-RICH REPEAT RECEPTOR PROTEIN KINASE EMS1,” a gene that plays critical roles in pathogenic defense responses (Gou *et al.* 2010).

#### Spectral indices BLUPs

**Fig. 9.**
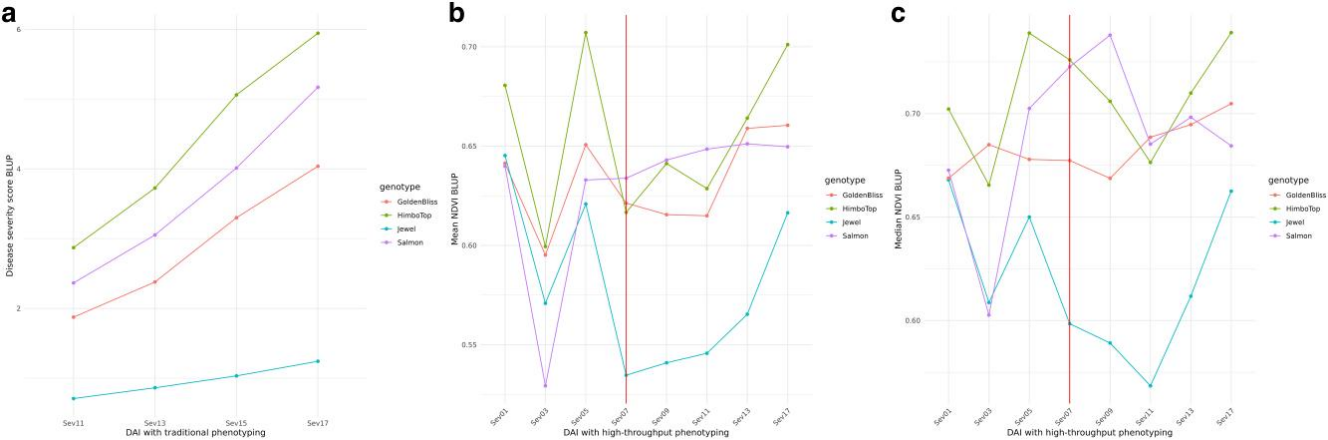
Disease severity BLUPs for the parents throughout DAI. a) The graph for traditional phenotyping, b) the graph for the mean NDVI index (high-throughput phenotyping), and c) the graph for the median NDVI. From the red vertical line (07 DAI onwards) symptoms began to appear in the inoculated plants.

As depicted in Fig. 9, the progression of the disease can be observed with the severity scores. Furthermore, we can observe the difference in parental resistance, with the “Jewel” variety (in blue) having the lowest disease severity values (Fig. 9a). The mean and median NDVI spectral indices at the 11th DAI (index within the highest correlation within traditional traits) appear random until the red line, which represents the day the plants began to show disease symptoms (Fig. 9b, c). From 7 DAI, there is a same pattern of disease severity behavior in parents, both for traditional and high-throughput phenotyping. The similarity of parental behavior in both phenotyping approaches suggests that we were also able to capture disease progression with high-throughput phenotyping. Furthermore, the index using the mean pixel value (Fig. 9b) produces a more similar pattern to the traditional phenotyping (Fig. 9a), than the index using the median pixel value (Fig. 9c).

## Discussion

### The polygenic architecture of late rust resistance

We aimed to understand the genetic architecture regulating resistance to late leaf rust in raspberries using traditional and high-throughput phenotyping, using statistical techniques such as GWAS and copula graphical models. The GWAS results showed a higher effect marker on chromosome 5 (Fig. 5). Still, there is evidence through copula graphical models that this trait can be regulated, not only by this higher effect marker but also for different genomic regions depending on the stage of disease progression (Fig. 7). Studies using other crops in the literature show that rust resistance is polygenic. Ledesma-Ramírez *et al.* (2019) found 14 resistance-associated SNPs to yellow rust in wheat, explaining 6.0–14.1% of the resistance. Vikas *et al.* (2022) found 65 QTNs associated with leaf rust resistance in bread wheat, explaining 1.98–31.72% of the phenotypic variation. We only found 1 associated marker in GWAS, which explains 43.13% of the phenotypic variation in 13 DAI (Table 2, SRO05_13334757 marker). Although this marker is a good candidate to carry out marker-assisted selection in raspberry breeding, we emphasize that the experiment has a small population and the effect of this marker may have been inflated for this same reason.

We believe the small population used is also why we did not find other SNPs. Although we knew that a larger population would be ideal, we lost many materials because the experimentation site is located in a subtropical region of Brazil. In contrast, the production sites have a predominantly temperate climate. In addition to the climatic difficulty, the interspecific hybrids’ vigor was unknown. Even with the reduced population, the significant marker found by GWAS has 9 candidate defense-related genes, in addition to other possible regions found by the network (Fig. 8). Clustering genes participating in the same metabolic pathway implies they are under the same selective pressure (Polturak and Osbourn 2021). Possibly, not just one of these genes confers resistance to raspberry, but others from the same cluster for a coordinated expression of specialized metabolites (Bharadwaj *et al.* 2021).

### Resistance to late rust possibly has a locus of higher effect

The higher effect marker for both the network and the GWAS had 4 candidate resistance genes flanking the marker. Resistance genes are involved in recognizing molecular patterns and elicitors of pathogens and are responsible for triggering the immune response in plants as soon as this recognition occurs. The “NB-ARC DOMAIN DISEASE RESISTANCE PROTEIN” gene, ∼20 kb from the marker, is a specific type of resistance gene that monitors intracellular disturbances related to plant immunity (van Ooijen *et al.* 2008). The “PLANT BROAD-SPECTRUM MILDEW RESISTANCE PROTEIN RPW8” gene was located at the exact location on the marker. RPW8 locus found in *Arabdopsis thaliana* contains a dominant resistance gene, which induces the salicylic acid metabolic pathway to trigger defense against fungal disease and powdery mildew (Xiao *et al.* 2001). The “WALL-ASSOCIATED RECEPTOR KINASE-LIKE 2” gene is 6.8 kb from the marker. WAKs, or Plant cell wall-associated kinases, are genes in many plant species that bind to cell wall pectin. WAKs also have an intracellular domain for signal transduction and are related to the recognition of damage-associated molecular pattern (DAMP), as cell wall fragments. By recognizing wall integrity, these receptors can trigger callose deposition on the cell wall and increase protection against pathogens (Amsbury 2020). The only work studying late rust resistance in raspberry was the (Jamieson and Nickerson 1999) work, in which they experimented with segregating populations for late rust resistance. They concluded that there is a locus with a higher effect on resistance called “Pa” by them, agreeing with our results.

### Gene flow throughout phenotyping days

An unexpected result was that, although both techniques have the SRO05_13334757 marker with higher association to late rust resistance with traditional phenotyping, only for GWAS does this same marker appear with a higher −log10(*P*-value) in all traits. Meanwhile, the other phenotyping days in the network, Sev11, Sev15, and Sev17, are more correlated with other markers. Possibly, the relationship between graph separation and conditional independence between variables showed us these differences in the network, as we consider all variables (traits and SNPs) simultaneously, not as a univariate test as in GWAS. Dias *et al.* (2023b) demonstrated that although the common parent has pre and postformed defense mechanisms against *A. americanum*, these are not sufficient to completely contain the infection. In Fig. 9a–c, although “Jewel” variety is more resistant than the other parents, its severity score also increases over time. With the intense inoculum application to all the leaves and more than 2 weeks of disease progression, the disease severity scores could change the genotype ranking on phenotyping days, as disease progression may not be the same for all genotypes. To understand whether the change in SNPs associated with each day of phenotyping is related to the type of modeling in the network or whether disease progression actually involves other genomic regions for resistance, could be a valuable information on late leaf rust resistance. But as we have a small population to infer this and these SNPs with lower effect in the network could be due to low statistical power, the answer is not within the scope of our study.

### A guidance for raspberries breeding

There are several studies demonstrating the usefulness of spectral indices in the diseased plants evaluation (Su *et al.* 2018; Abdullah *et al.* 2022; Soares *et al.* 2022). Thus, our objective was to test the usefulness of the NDVI and OSAVI indices for high-throughput phenotyping for late rust resistance in raspberry breeding. However, the markers found are different between the phenotyping approaches and the correlation of spectral indices with traditional phenotypes only reached 0.26. We attribute this low correlation to some factors that we noticed throughout the experiments. The first is that young raspberry leaves are resistant to late rust and the images were taken from the top of the plants, capturing mostly young leaves. During the experiments, we noticed an intense vegetative growth, but as the genotypes had different growth rates and as we did not want to cause stress to the plants, we did not prune them. Meanwhile, traditional phenotyping consisted of a general score based in the old leaves. The second possible reason is that, we noticed that the common and resistant parent showed symptoms on the leaves adaxial (upper) face in the first few days of plant symptoms. As the disease progressed, the common parent did not develop the characteristic orange pustules on the abaxial (lower) face, meaning the pathogen was able to infect the common parent but was unable to continue its reproductive cycle and develop spores (Supplementary Fig. S2). Figure 9a shows that traditional severity scores for the common parent were maintained close to zero during the 17 DAI. In this way, the spectral indices may have been distanced from the traditional scores because the latter took pustules into account when assigning a severity score, while the former only used images of the upper part of the plants where the pustules are not located. The third and final reason is that the NDVI and OSAVI indices use spectral bands outside the visible spectrum (Su *et al.* 2018), such as Near Infrared, which may result in differences in relation to traditional phenotypes. However, even with these difficulties encountered during the experiment, the spectral indices were still able to capture the disease progression in the different parental lines (Fig. 9). Furthermore, the significant marker found on chromosome 4, with the mean NDVI index at 11 DAI, has a resistance gene close to it (40 kb away), which could potentially be an important marker for resistance to late rust and a potential way of phenotyping plants in an automated way, as long as it is improved to deal with the biological challenges encountered by us.

Although our work has offered different confidence levels with the obtained results, it has produced important information for directing raspberry breeding programs. It was possible to indicate the marker with the highest effect in one of the main crop diseases using different statistical approaches; both led to consistent results, supporting our findings and conclusions. Furthermore, using a GWAS/copula graphical models pipeline, which is not conventional in plant breeding, can help other researchers discover causal alleles for their study traits. Using this pipeline, we could also add other candidate genes with less effect but still possibly responsible for the same trait. Therefore, a natural progression for this work would be to carry out validation studies of the markers found to confer reproducibility and consistency, as no other works are studying the genetic control of late leaf rust in raspberries to compare significant markers.

## Supplementary Material

jkae202_Supplementary_Data

## Data Availability

Genomic data prior to filtering in Variant Call Format (VCF) is available in the Rosaceae database, under the accession number tfGDR1071, under the name “Rocci_raw_imput_all_sort.vcf” and can be accessed with the https://www.rosaceae.org/publication_datasets URL. Traditional phenotypes (Supplementary Table S4), high-throughput phenotypes (Supplementary Table S5), and the table of markers after filtering (Supplementary Table S6) are available as Supplementary material. Alternatively, these data can be downloaded directly from the “RaspberryRust” repository on GitHub (https://github.com/MelinaPrado/RaspberryRust.git). Supplemental material available at G3 online.

## References

[jkae202-B1] Abdullah AA, Arnous MO, Bayoumi T, Nabi HMAE. 2022. Utilizing hyper-spectral no-image measurement to assess the development of disease severity of Cercospora Leaf Spot disease in sugar beet canopy. J Plant Prod Sci. 11:37–45. doi:10.21608/jpps.2022.234798.

[jkae202-B2] Alonso-Blanco C, Aarts MG, Bentsink L, Keurentjes JJ, Reymond M, Vreugdenhil D, Koornneef M. 2009. What has natural variation taught us about plant development, physiology, and adaptation? Plant Cell. 21(7):1877–1896. doi:10.1105/tpc.109.068114 19574434 PMC2729614

[jkae202-B3] Amsbury S . 2020. Sensing attack: the role of wall-associated kinases in plant pathogen responses. Plant Physiol. 183:1420–1421. doi:10.1104/pp.20.00821 32747491 PMC7401100

[jkae202-B4] Araus JL, Kefauver SC, Zaman-Allah M, Olsen MS, Cairns JE. 2018. Translating high-throughput phenotyping into genetic gain. Trends Plant Sci. 23(5):451–466. doi:10.1016/j.tplants.2018.02.001 29555431 PMC5931794

[jkae202-B5] Baby B, Antony P, Vijayan R. 2018. Antioxidant and anticancer properties of berries. Crit Rev Food Sci Nutr. 58(15):2491–2507. doi:10.1080/10408398.2017.1329198 28609132

[jkae202-B6] Behrouzi P, Arends D, Wit EC. 2023. The R journal: Netgwas: an R package for network-based genome wide association studies. R J. 14:18–37. doi:10.32614/RJ-2023-011

[jkae202-B7] Behrouzi P, Wit EC. 2019. Detecting epistatic selection with partially observed genotype data by using copula graphical models. J R Stat Soc Ser C (Appl Stat). 68(1):141–160. doi:10.1111/rssc.12287

[jkae202-B8] Bharadwaj R, Kumar SR, Sharma A, Sathishkumar R. 2021. Plant metabolic gene clusters: evolution, organization, and their applications in synthetic biology. Front Plant Sci. 12:1–23. doi:10.3389/fpls.2021.697318 PMC8418127 34490002

[jkae202-B9] Campos GR, Prado M, Reis Borges KL, Yassue RM, Sabadin F, da Silva AV, Morais de Alcântara Barbosa C, Bellato Sposito M, Amorim L, Fritsche-Neto R. 2023. Construction and genetic characterization of an interspecific raspberry hybrids panel aiming resistance to late leaf rust and adaptation to tropical regions. Sci Rep. 13(1):15216. doi:10.1038/s41598-023-41728-8 37709795 PMC10502132

[jkae202-B10] Chen ZQ, Zan Y, Milesi P, Zhou L, Chen J, Li L, Cui B, Niu S, Westin J, Karlsson B, *et al*. 2021. Leveraging breeding programs and genomic data in Norway spruce (*Picea abies* L. Karst) for GWAS analysis. Genome Biol. 22(1):179. doi:10.1186/s13059-021-02392-1 34120648 PMC8201819

[jkae202-B11] de Jongh R, Behrouzi P. 2022. *netShiny: Tool for Comparison and Visualization of Multiple Networks*. R package version 1.0

[jkae202-B12] de Oliveira JR, Silva JVG, Amourim MAA, Santos MN, Batista AG. 2020. Producao de pequenas frutas no Brasil: um mercado em potencial. Encic Bios. 17:362–380.

[jkae202-B13] Dias MG, Ribeiro RR, de A Barbosa CM, de Jesus JMI, Spósito MB. 2023a. Diagrammatic scale for improved late leaf rust severity assessments in raspberry leaves. Can J Plant Pathol. 45(2):140–147. doi:10.1080/07060661.2022.2147587

[jkae202-B14] Dias MG, Spósito MB, Tessmer MA, Appezzato-da Glória B. 2023b. Investigating biochemical and histopathological responses between raspberries and *Aculeastrum americanum*. J Fungi. 9(3):337. doi:10.3390/jof9030337 PMC10054533 36983505

[jkae202-B15] Durante F, Fernández-Sánchez J, Sempi C. 2013. A topological proof of Sklar’s theorem. Appl Math Lett. 26(9):945–948. doi:10.1016/j.aml.2013.04.005

[jkae202-B16] Ellis MA, Converse RH, Williams RN, Williamson B. 1991. Compendium of Raspberry and Blackberry Diseases and Insects. 2nd ed. St. Paul (MN): APS PRESS.

[jkae202-B17] Elshire RJ, Glaubitz JC, Sun Q, Poland JA, Kawamoto K, Buckler ES, Mitchell SE. 2011. A robust, simple genotyping-by-sequencing (GBS) approach for high diversity species. PLoS One. 6(5):e19379. doi:10.1371/journal.pone.0019379 21573248 PMC3087801

[jkae202-B18] FAOSTAT . Food and Agriculture Organization of the United Nations. Crops and livestock products. FAOSTAT database. https://www.fao.org/faostat/en/#data. 2022.

[jkae202-B19] Folta K. M., Gardiner S. E. 2009. Genetics and genomics of Rosaceae. New York: Springer. p. 507–524.

[jkae202-B20] Gill T, Gill SK, Saini DK, Chopra Y, de Koff JP, Sandhu KS. 2022. A comprehensive review of high throughput phenotyping and machine learning for plant stress phenotyping. Phenomics. 2:156–183. doi:10.1007/s43657-022-00048-z 36939773 PMC9590503

[jkae202-B21] Glaubitz JC, Casstevens TM, Lu F, Harriman J, Elshire RJ, Sun Q, Buckler ES. 2014. TASSEL-GBS: a high capacity genotyping by sequencing analysis pipeline. PLoS One. 9(2):e90346. doi:10.1371/journal.pone.0090346 24587335 PMC3938676

[jkae202-B22] Gou X, He K, Yang H, Yuan T, Lin H, Clouse SD, Li J. 2010. Genome-wide cloning and sequence analysis of leucine-rich repeat receptor-like protein kinase genes in *Arabidopsis thaliana*. BMC Genomics. 11(1):19. doi:10.1186/1471-2164-11-19 20064227 PMC2817689

[jkae202-B23] Granato ISC, Galli G, de Oliveira Couto EG, e Souza MB, Mendonça LF, Fritsche-Neto R. 2018. snpReady: a tool to assist breeders in genomic analysis. Mol Breed. 38(8):102. doi:10.1007/s11032-018-0844-8

[jkae202-B24] Hall HK, Hummer KE, Jamieson AR, Jennings SN, Weber CA. 2009. Plant Breeding Reviews. Hoboken (NJ): Willey-Blackwell.

[jkae202-B25] Hauenstein H. Variety of raspberry plant named “RAFZAOU”. U.S. Patent and Trademark Office US (PP19,512 P3). 2008.

[jkae202-B26] Henson J, Tischler G, Ning Z. 2012. Next-generation sequencing and large genome assemblies. Pharmacogenomics. 13(8):901–915. doi:10.2217/pgs.12.72 22676195 PMC3960634

[jkae202-B27] Huang X, Han B. 2014. Natural variations and genome-wide association studies in crop plants. Annu Rev Plant Biol. 65:531–551. doi:10.1146/arplant.2014.65.issue-1 24274033

[jkae202-B28] Jamieson AR, Nickerson NL. 1999. Inheritance of resistance to late yellow rust (*Pucciniastrum americanum*) in red raspberry. Acta Hortic. 505(505):53–58. doi:10.17660/ActaHortic.1999.505.5

[jkae202-B29] Johnson RC, Nelson GW, Troyer JL, Lautenberger JA, Kessing BD, Winkler CA, O’Brien SJ. 2010. Accounting for multiple comparisons in a genome-wide association study (GWAS). BMC Genomics. 11(1):724. doi:10.1186/1471-2164-11-724 21176216 PMC3023815

[jkae202-B30] Korte A, Farlow A. 2013. The advantages and limitations of trait analysis with GWAS: a review. Plant Methods. 9:1–9. doi:10.1186/1746-4811-9-29 23876160 PMC3750305

[jkae202-B31] Langmead B, Salzberg SL. 2012. Fast gapped-read alignment with Bowtie 2. Nat Methods. 9(4):357–359. doi:10.1038/nmeth.1923 22388286 PMC3322381

[jkae202-B32] Ledesma-Ramírez L, Solís-Moya E, Iturriaga G, Sehgal D, Reyes-Valdes MH, Montero-Tavera V, Sansaloni CP, Burgueño J, Ortiz C, Aguirre-Mancilla CL. 2019. GWAS to identify genetic loci for resistance to yellow rust in wheat pre-breeding lines derived from diverse exotic crosses. Front Plant Sci. 10:1–14. 10.3389/fpls.2019.01390 31781137 PMC6831551

[jkae202-B33] Lewis D, Crowe LK. 1958. Unilateral interspecific incompatibility in flowering plants. Heredity. 24. doi:10.1038/hdy.1958.26

[jkae202-B34] Lipka AE, Tian F, Wang Q, Peiffer J, Li M, Bradbury PJ, Gore MA, Buckler ES, Zhang Z. 2012. GAPIT: genome association and prediction integrated tool. Bioinformatics. 28:2397–2399. doi:10.1093/bioinformatics/bts444 22796960

[jkae202-B35] Marchi PM, Carvalho IR, Pereira IdS, Rosa TCd, Höhn D, Szareski VJ, Reisser C, Antunes LEC. 2019. Yield and quality of primocane-fruiting raspberry grown under plastic cover in southern Brazil. Sci Agric. 76(6):481–486. doi:10.1590/1678-992x-2018-0154

[jkae202-B36] Matias FI, Caraza-Harter MV, Endelman JB. 2020. FIELDimageR: an R package to analyze orthomosaic images from agricultural field trials. Plant Phenome J. 3:e20005. doi:10.1002/ppj2.20005

[jkae202-B37] Moura PHA, Campagnolo MA, Pio R, Curi PN, Assis CND, Silva TC. 2012. Fenologia e produção de cultivares de framboeseiras em regiões subtropicais no Brasil. Pesqui Agropecu Bras. 47(12):1714–1721. doi:10.1590/S0100-204X2012001200006

[jkae202-B38] OMAFRA . 2021. *Ontario Ministry of Agriculture, Food and Rural Affairs - Raspberry Variety Description*. https://www.ontario.ca/page/raspberry-variety-description.

[jkae202-B39] Ourecky DK, Slate GL. 1973. The New York raspberry breeding program. Geneva (NY): New York State Agricultural Experiment Station.

[jkae202-B40] Polturak G, Osbourn A. 2021. The emerging role of biosynthetic gene clusters in plant defense and plant interactions. PLoS Pathog. 17:e1009698. doi:10.1371/journal.ppat.1009698 34214143 PMC8253395

[jkae202-B41] Purutçuoğlu V, Farnoudkia H. 2017. Copula Gaussian graphical modeling of biological networks and Bayesian inference of model parameters. Sci Iran. 26:2495–2505. doi:10.24200/sci.2019.5071.1076

[jkae202-B42] Rao AV, Snyder DM. 2010. Raspberries and human health: a review. J Agric Food Chem. 58:3871–3883. doi:10.1021/jf903484g 20178390

[jkae202-B43] Raseira M, Gonçalves E, Trevisa R, Antunes L, Aspectos técnicos da cultura da framboeseira. Embrapa Clima Temperado. 2004.

[jkae202-B44] Rodríguez-Álvarez MX, Boer MP, van Eeuwijk FA, Eilers PH. 2017. Correcting for spatial heterogeneity in plant breeding experiments with p-splines. Spat Stat. 23:52–71. doi:10.1016/j.spasta.2017.10.003

[jkae202-B45] Rossum BJv, Eeuwijk FAV, Boer M, Malosetti M, Bustos-Korts D, Millet E, Paulo J. 2023. *statgenSTA: Single Trial Analysis (STA) of Field Trials*. R package version 1.0.11

[jkae202-B46] Soares AdS, Vieira BS, Bezerra TA, Martins GD, Siquieroli ACS. 2022. Early detection of coffee leaf rust caused by *Hemileia vastatrix* using multispectral images. Agronomy. 12:2911. doi:10.3390/agronomy12122911

[jkae202-B47] Su J, Liu C, Coombes M, Hu X, Wang C, Xu X, Li Q, Guo L, Chen WH. 2018. Wheat yellow rust monitoring by learning from multispectral UAV aerial imagery. Comput Electron Agric. 155:157–166. doi:10.1016/j.compag.2018.10.017

[jkae202-B48] VanBuren R, Bryant D, Bushakra JM, Vining KJ, Edger PP, Rowley ER, Priest HD, Michael TP, Lyons E, Filichkin SA, *et al*. 2016. The genome of black raspberry (*Rubus occidentalis*). Plant J Cell Mol Biol. 87(6):535–547. doi:10.1111/tpj.2016.87.issue-6 27228578

[jkae202-B49] van Ooijen G, Mayr G, Kasiem MMA, Albrecht M, Cornelissen BJC, Takken FLW. 2008. Structure-function analysis of the NB-ARC domain of plant disease resistance proteins. J Exp Bot. 59:1383–1397. doi:10.1093/jxb/ern045 18390848

[jkae202-B50] Vikas VK, Pradhan AK, Budhlakoti N, Mishra DC, Chandra T, Bhardwaj SC, Kumar S, Sivasamy M, Jayaprakash P, Nisha R, *et al*. 2022. Multi-locus genome-wide association studies (ML-GWAS) reveal novel genomic regions associated with seedling and adult plant stage leaf rust resistance in bread wheat (*Triticum aestivum* L.). Heredity. 128(6):434–449. doi:10.1038/s41437-022-00525-1 35418669 PMC9177675

[jkae202-B51] Ward JA, Bhangoo J, Fernández-Fernández F, Moore P, Swanson J, Viola R, Velasco R, Bassil N, Weber CA, Sargent DJ. 2013. Saturated linkage map construction in *Rubus idaeus* using genotyping by sequencing and genome-independent imputation. BMC Genomics. 14(1):2. doi:10.1186/1471-2164-14-2 23324311 PMC3575332

[jkae202-B52] Willman MR, Bushakra JM, Bassil N, Finn CE, Dossett M, Perkins-Veazie P, Bradish CM, Fernandez GE, Weber CA, Scheerens JC, *et al*. 2022. Analysis of a multi-environment trial for black raspberry (*Rubus occidentalis* L.) quality traits. Genes. 13(3):418. doi:10.3390/genes13030418 35327972 PMC8950803

[jkae202-B53] Xiao S, Ellwood S, Calis O, Patrick E, Li T, Coleman M, Turner JG. 2001. Broad-spectrum mildew resistance in *Arabidopsis thaliana* mediated by RPW8. Science. 291(5501):118–120. doi:10.1126/science.291.5501.118 11141561

[jkae202-B54] Yang W, Feng H, Zhang X, Zhang J, Doonan JH, Batchelor WD, Xiong L, Yan J. 2020. Crop phenomics and high-throughput phenotyping: past decades, current challenges, and future perspectives. Mol Plant. 13:187–214. doi:10.1016/j.molp.2020.01.008 31981735

[jkae202-B55] Yassue RM, Galli G, Junior RB, Cheng H, Morota G, Fritsche-Neto R. 2022. A low-cost greenhouse-based high-throughput phenotyping platform for genetic studies: a case study in maize under inoculation with plant growth-promoting bacteria. Plant Phenome J. 5(1), e20043. doi:10.1002/ppj2.20043

[jkae202-B56] Zhang A, Fang J, Hu W, Calhoun VD, Wang YP. 2021. A latent Gaussian copula model for mixed data analysis in brain imaging genetics. IEEE/ACM Trans Comput Biol Bioinform. 18(4):1350–1360. doi:10.1109/TCBB.2019.2950904 31689199 PMC7756188

[jkae202-B57] Zheng X, Levine D, Li JZ, Weir SM, Willer CJ, Abecasis GR. 2023. *SNPRelate: Parallel Computing Toolset for Relatedness and Principal Component Analysis of SNP Data*. R package version 1.34.1.

