# Supplementary figures and images for: Complementary approaches to dissect late leaf rust resistance in an interspecific raspberry population
Source: G3 (Bethesda). 2024 Aug 22;14(10):jkae202. doi: 10.1093/g3journal/jkae202 (PMC11457092; doi:10.1093/g3journal/jkae202)

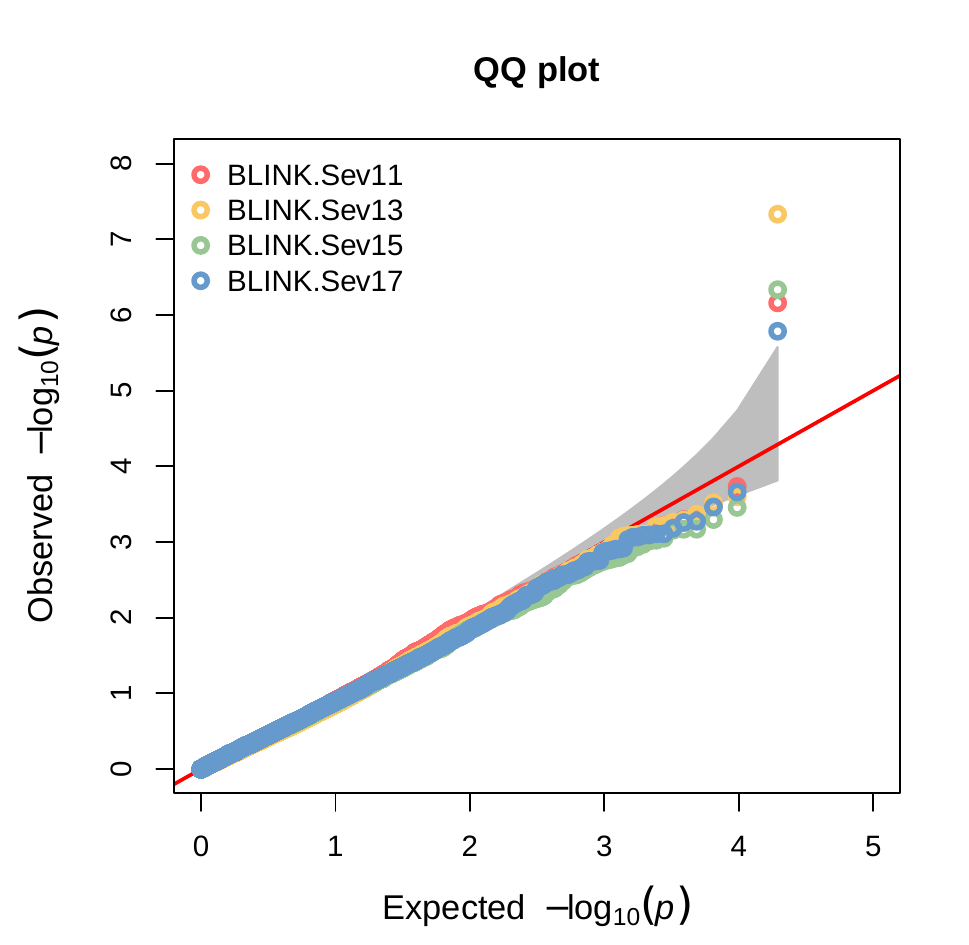

Supplement: jkae202_Supplementary_Data [file jkae202_supplementary_data.zip › Figure_S1_G3-2024-405231.tif]

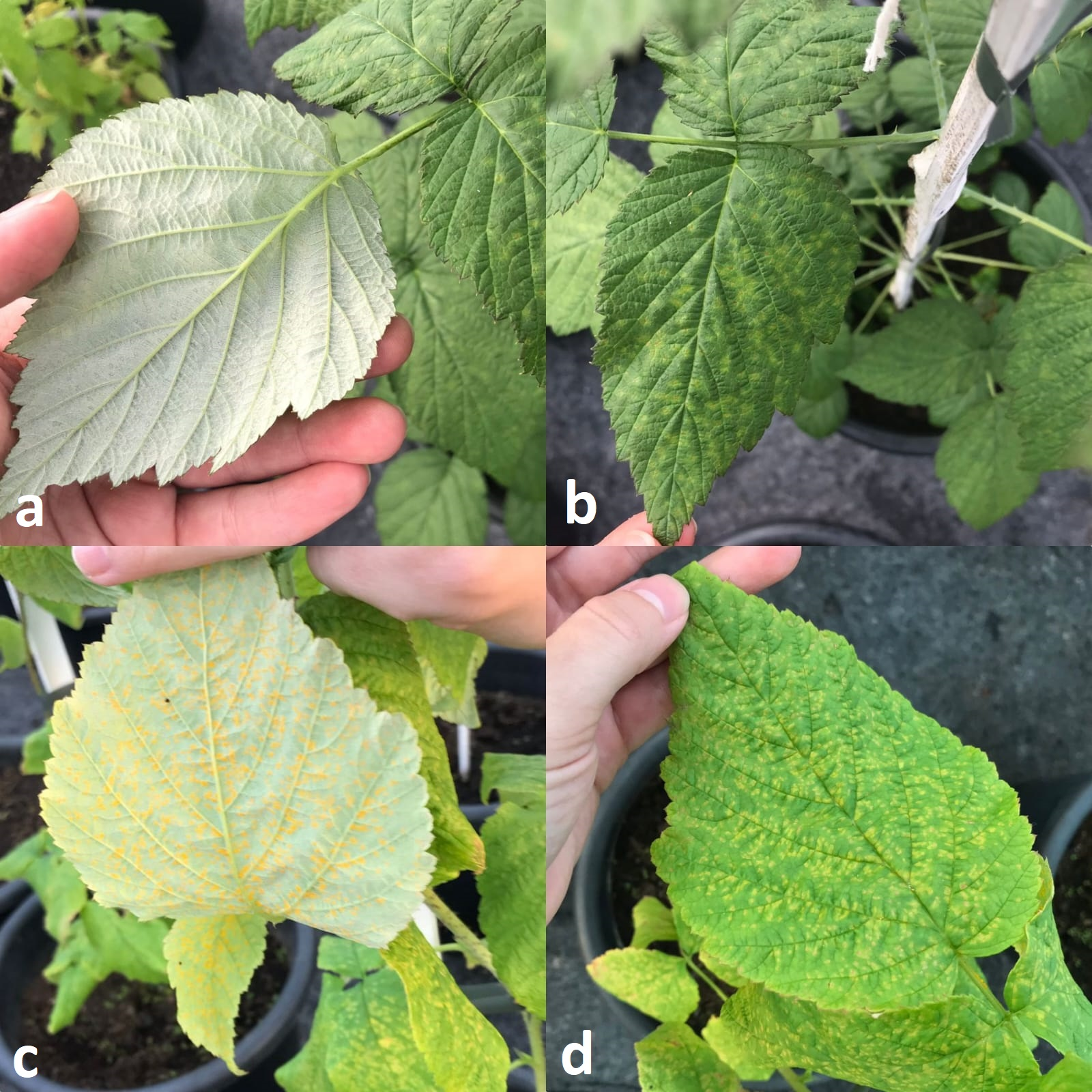

Supplement: jkae202_Supplementary_Data [file jkae202_supplementary_data.zip › Figure_S2_G3-2024-405231.tif]
